# Protective role for the N-terminal domain of α-dystroglycan in Influenza A virus proliferation

**DOI:** 10.1073/pnas.1904493116

**Published:** 2019-05-16

**Authors:** Jessica C. de Greef, Bram Slütter, Mary E. Anderson, Rebecca Hamlyn, Raul O’Campo Landa, Ellison J. McNutt, Yuji Hara, Lecia L. Pewe, David Venzke, Kiichiro Matsumura, Fumiaki Saito, John T. Harty, Kevin P. Campbell

**Affiliations:** ^a^Howard Hughes Medical Institute, The University of Iowa, Iowa City, IA 52242;; ^b^Department of Molecular Physiology and Biophysics, The University of Iowa, Iowa City, IA 52242;; ^c^Department of Neurology, The University of Iowa, Iowa City, IA 52242;; ^d^Department of Microbiology and Immunology, The University of Iowa, Iowa City, IA 52242;; ^e^Department of Neurology, Teikyo University School of Medicine, 173-8605 Tokyo, Japan;; ^f^Department of Pathology, The University of Iowa, Iowa City, IA 52242

**Keywords:** α-dystroglycan, influenza A virus, inflammation

## Abstract

α-Dystroglycan (α-DG) is a highly glycosylated basement membrane receptor that is cleaved by the proprotein convertase furin, which releases its N-terminal domain (α-DGN). Before cleavage, α-DGN interacts with the glycosyltransferase LARGE1 and initiates functional O-glycosylation of the mucin-like domain of α-DG. Notably, α-DGN has been detected in a wide variety of human bodily fluids, but the physiological significance of secreted α-DGN remains unknown. Here, we show that mice lacking α-DGN exhibit significantly higher viral titers in the lungs after Influenza A virus (IAV) infection (strain A/Puerto Rico/8/1934 H1N1), suggesting an inability to control virus load. Consistent with this, overexpression of α-DGN before infection or intranasal treatment with recombinant α-DGN prior and during infection, significantly reduced IAV titers in the lungs of wild-type mice. Hemagglutination inhibition assays using recombinant α-DGN showed in vitro neutralization of IAV. Collectively, our results support a protective role for α-DGN in IAV proliferation.

## Significance

Influenza A virus (IAV) is a major cause of respiratory infections. We show that mice lacking the N-terminal domain of α-dystroglycan (α-DGN) exhibit significantly higher viral titers in the lungs after IAV infection. In addition, we show that overexpression of α-DGN in the lungs, both prior and during IAV infection, significantly reduces viral load and that recombinant α-DGN disrupts hemagglutination mediated by the influenza virus. Collectively, we uncover a protective role for α-DGN in IAV proliferation, suggesting it may have antiviral properties and could potentially be used as a treatment for IAV infection. As α-DGN levels are altered in more (inflammatory) disease states, this insight opens new avenues of investigation into the role of α-DGN in inflammation.

The membrane receptor dystroglycan (DG) links the extracellular matrix to the cytoskeleton in many cell types (1–4). As such, DG has important functions in many processes, including basement membrane assembly (5) and muscle regeneration (6). Further, it acts as a receptor for lymphocytic choriomeningitis virus and Lassa fever virus, which facilitates viral infection (7). DG is encoded by the *DAG1* gene, and the resulting polypeptide is cleaved into α-DG and β-DG by autoproteolysis (2, 8). β-DG is the transmembrane subunit and it interacts noncovalently with α-DG; its intracellular domain binds to the cytoskeletal protein dystrophin (2, 3, 9, 10). α-DG is the extracellular subunit that binds to extracellular matrix proteins including agrin, laminin, neurexin, perlecan, and pickachurin (2, 11–14). The ability of α-DG to bind to extracellular matrix proteins depends on posttranslational modifications, specifically O-glycosylation of its mucin-like domain (amino acids 313–485) (15, 16). This also requires the N-terminal domain of α-DG (α-DGN; amino acids 1–312), which interacts with the glycosyltransferase LARGE1 to initiate functional O-glycosylation of the mucin-like domain (17, 18). When α-DG lacks α-DGN, interaction with LARGE1 is impaired and the function of α-DG as an extracellular matrix receptor is abolished (17).

Several studies indicate a role for α-DGN beyond functional O-glycosylation of α-DG. α-DGN is naturally cleaved by the proprotein convertase furin at the sequence RVRR (amino acids 309–312) (19), which does not disturb the function of α-DG (17). α-DGN has a globular structure that is organized into two subdomains (20). The first subdomain is a typical Ig-like domain; the second subdomain resembles ribosomal RNA-binding proteins (21). Notably, α-DGN is secreted by cells in culture (22) and has been detected in a wide variety of human bodily fluids, including serum and plasma (22, 23), urine (24), cerebrospinal fluid (22, 24), and lachrymal fluid (24). One study showed that recombinant α-DGN binds to several laminins, fibronectin, and fibrinogen in regenerating peripheral nerves, and that the addition of recombinant α-DGN to PC12 cells promotes neurite extension (25). Interestingly, increased α-DGN levels have been observed in the cerebrospinal fluid of patients with Lyme neuroborreliosis (24) and in the uterine lavage of early-stage endometrial cancer patients (26). The latter study suggests a role for α-DGN in maintaining the integrity of tight junctions and the polarity of endometrial epithelial cells. In contrast, decreased levels of α-DGN have been reported in the serum of patients with Duchenne muscular dystrophy (DMD) and in the serum of utrophin-deficient *mdx* mice, a mouse model for DMD (27). Thus, α-DGN levels are altered in certain disease states, yet the functional significance of its secreted form remains largely unknown.

In line with this, expression of *furin*, which can potentially affect the secretion of α-DGN, is also changed in specific disorders, including in several inflammatory disorders. For example, elevated *furin* expression levels have been observed in various types of cancer (28) and furin protein has been found in atherosclerotic plaques (29). In addition, *furin* is up-regulated in activated T cells (30) and is highly expressed in samples from rheumatoid arthritis patients (31). Given the potential for furin to affect the secretion of α-DGN, and the altered expression of both α-DGN and furin in the context of disease, we hypothesized that inflammation induces *furin* expression leading to increased secretion of α-DGN. To address this hypothesis, we studied *furin* expression and α-DGN secretion in the context of Influenza A virus (IAV) infection, which causes a localized respiratory infection associated with inflammation in the airways. We show that in the context of IAV infection, *furin* expression is increased and that secreted α-DGN is detectable in murine airways. In addition, mice lacking α-DGN exhibit significantly higher viral titers in whole lung tissue after IAV infection, while overexpression of α-DGN in the lungs significantly reduced viral load after IAV infection, suggesting that α-DGN plays a role in the clearance of IAV and may have antiviral properties. In conclusion, we show an unexpected role for α-DGN that goes beyond its function as an interacting partner of LARGE1.

## Results

### Infection with IAV Elevates *Furin* Expression in the Lungs and Decreases α-DGN Levels in the Bronchoalveolar Lavage Fluid.

**Fig. 1.**
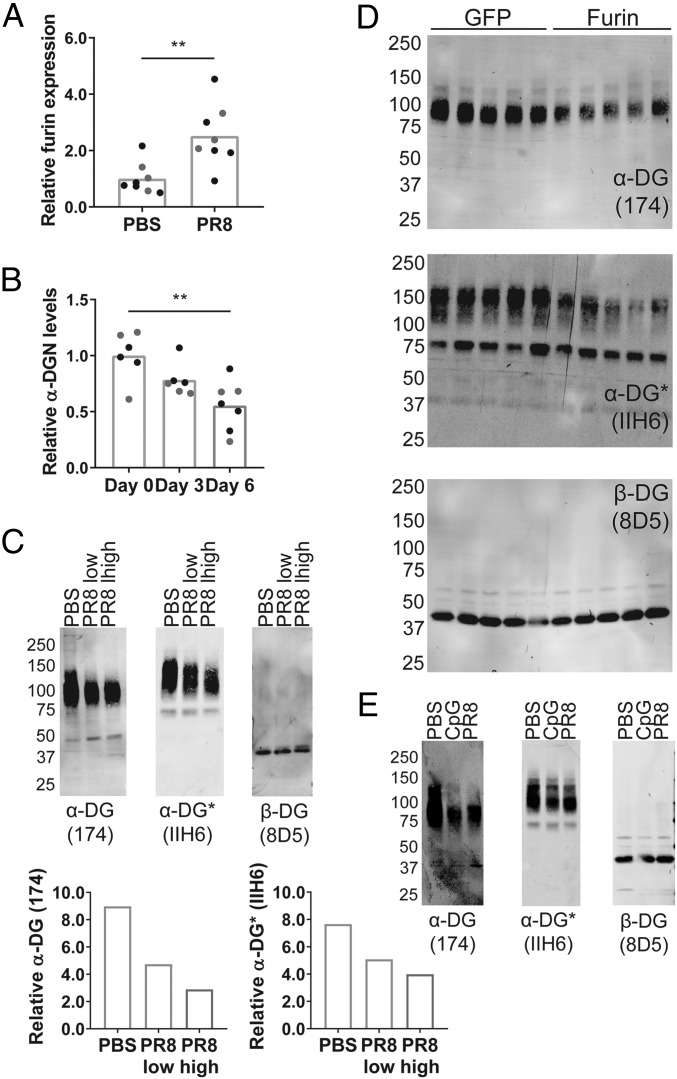
Infection with IAV elevates furin expression in the lungs and decreases α-DGN levels in the BAL fluid. (*A*) *Furin* expression in whole lung tissue of PR8-infected C57BL/6 mice 6 d after infection. *Furin* expression is relative to the housekeeping genes *Rpl4* and *Rps29* and normalized to expression in PBS-treated C57BL/6 mice, which was set at 1. Statistical analysis was performed using a Student’s *t* test. (*B*) Relative α-DGN levels in the BAL fluid of PR8-infected C57BL/6 mice at the indicated day following infection. Data are normalized to the α-DGN concentration on day 0, which was set at 1. Statistical analysis was performed using Kruskal–Wallis analysis of variance on ranks followed by Dunn’s multiple comparison tests. Data consist of two independent experiments with three to five mice (*A*) or three to four mice (*B*) per group in each experiment. Each dot represents an individual mouse; black dots represent mice in experiment 1, gray dots represent mice in experiment 2. **P < 0.01 (*A* and *B*). (*C*–*E*) α-DG protein expression, α-DG glycosylation levels, and β-DG protein expression in whole lung tissue from PR8-infected C57BL/6 mice 6 d after IAV infection (*C*), 2 wk after infection with an adenovirus encoding furin (*D*), or 1 d after intranasal treatment with CpG ODN (*E*). Mice received PBS, a low dose of PR8 (1 × 10^5^ TCID_50_; PR8 low), or a higher dose of PR8 (2 × 10^5^ TCID_50_; PR8 high). α-DG protein expression and α-DG glycosylation levels were quantified relative to β-DG protein expression (*C*). Mice received a control GFP-expressing adenovirus (GFP) or a furin-expressing adenovirus (Furin) (*D*). Mice received PBS, CpG ODN (on two occasions, 3 d apart; CpG), or PR8 (2 × 10^5^ TCID_50_; PR8). Mice that received CpG ODN were killed 1 d after the second treatment; mice that received intranasal PBS or PR8 were killed on day 6 after infection (*E*). Data are representative of three (*C*) or two independent experiments (*D* and *E*). Each lane represents an individual mouse. Protein size indicated is in kilodaltons. The Sheep174 antibody binds to α-DG even in the absence of glycosylation. The IIH6 antibody detects the glycosylated form of α-DG (*). The 8D5 antibody detects β-DG (*C*–*E*).

To test our hypothesis that inflammation induces *furin* expression, which leads to increased secretion of α-DGN, we infected wild-type C57BL/6 mice with the mouse-adapted IAV strain A/Puerto Rico/8/1934 H1N1 (PR8). Using quantitative RT-PCR, we next detected a significant increase in the expression of *furin* in whole lung tissue of PR8-infected C57BL/6 mice, but not in whole lung tissue of C57BL/6 mice administered phosphate-buffered saline (PBS), on day 6 after infection [*P* = 0.0034, Fig. 1*A* (relative data), *SI Appendix*, Fig. S1 (absolute data)], indicating that inflammation indeed induces *furin* expression. To determine if the increased expression of furin is associated with an increase in α-DGN secretion, we assessed the levels of α-DGN in the bronchoalveolar lavage (BAL) fluid of C57BL/6 mice that were infected with PR8 using an ELISA. For this assay, we used the α-DGN antibody Sheep173, an affinity-purified sheep polyclonal antibody that was made using the complete N-terminal region of α-DG (*SI Appendix*, Fig. S2). Surprisingly, we detected a significant decrease in the level of secreted α-DGN in the BAL fluid of these mice [*P* = 0.0030, Fig. 1*B* (relative data), *SI Appendix*, Fig. S3 (absolute data)]. However, intranasal PR8 infection resulted in a dose-dependent reduction of α-DG glycosylation levels in whole lung tissue of C57BL/6 mice, as detected by Western blot analysis using the IIH6 antibody that detects the glycosylated form of α-DG, without altering the amount of detectable β-DG (Fig. 1*C*). As α-DGN cleavage may affect the interaction between α-DG and LARGE1 and, thus, glycosylation of α-DG, these results suggest that the elevated expression of *furin* indeed led to accelerated cleavage of α-DGN from α-DG.

To confirm this, we infected C57BL/6 mice intranasally with an adenovirus to overexpress furin. Two weeks later, we measured reduced α-DG glycosylation levels in whole lung tissue of these mice (Fig. 1*D*). Next, to assess α-DG glycosylation levels in a situation that mimics the pulmonary inflammatory response that is observed after PR8 infection, we administered intranasal CpG oligonucleotides (ODN), a Toll-like receptor 9 agonist (32), in C57BL/6 mice. CpG ODN treatment also reduced α-DG glycosylation levels in the lungs (Fig. 1*E*), suggesting that α-DGN cleavage results from IAV-induced inflammation rather than from the infection of cells. Thus, despite detecting reduced levels of α-DGN in the BAL fluid after PR8 infection, we observed elevated expression of *furin* and reduced levels of α-DG glycosylation, suggesting increased secretion of α-DGN upon IAV infection. The reduced levels of α-DGN in the BAL fluid following PR8 infection suggest that it may have a function during IAV infection and is engaged in an, as of yet, unknown process.

### Mice that Lack DG or α-DGN Exhibit Higher Viral Titers in the Lungs After IAV Infection.

To further investigate a potential function for α-DGN during IAV infection, we next infected (*i*) mice that lack the complete *Dag1* gene, which encodes both α-DG and β-DG (DG-KO mice), and (*ii*) mice that lack the complete *Dag1* gene on one allele and lack the N-terminal domain of α-DG on the second allele (α-DGN-KO mice). Since glycosylation of α-DG is required for the formation of an extraembryonic basement membrane, the Reichert’s membrane (33), conventional DG-KO mice and α-DGN-KO mice exhibit an embryonic lethal phenotype. Thus, we used tamoxifen-inducible DG-KO mice and tamoxifen-inducible α-DGN-KO mice for our experiments; in both mouse models, genetic deletion can be induced in a time-specific manner upon administration of tamoxifen (34).

**Fig. 2.**
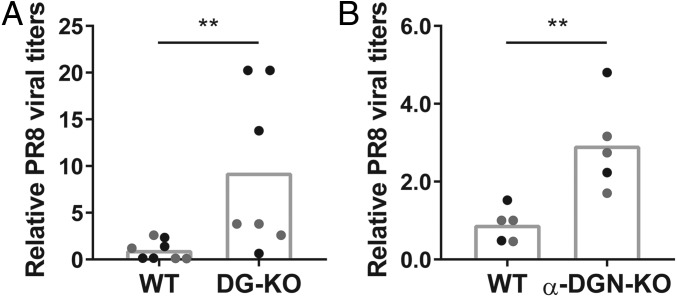
Mice that lack DG or α-DGN exhibit higher viral titers in the lungs after IAV infection. (*A* and *B*) Relative titers of PR8 virus on day 4 after infection in whole lung tissue of control (WT) mice and those that lack DG (DG-KO) (*A*) or α-DGN (α-DGN-KO) (*B*). Data consist of two independent experiments with three to four mice (*A*) or two to three mice (*B*) per group in each experiment. Titers from DG-KO mice (*A*) and α-DGN-KO mice (*B*) are relative to those from control mice, which were set at 1. Each dot represents an individual mouse: black dots represent mice in experiment 1; gray dots represent mice in experiment 2. Statistical analysis was performed using a Mann–Whitney *U* test. **P < 0.01 (*A* and *B*).

All mice first received two doses of tamoxifen at 2 mo of age by oral gavage. Cre-negative littermates that also received tamoxifen were used as control mice. Two months after administering tamoxifen, both Cre-positive mice and Cre-negative mice were infected with PR8. Four days following PR8 infection, the day that the highest viral titers in whole lung tissue can be measured (35), skeletal muscle was harvested to confirm gene deletion and whole lung tissue was obtained to determine viral titers. Skeletal muscle from these mice confirmed that tamoxifen treatment resulted in the loss of both α-DG protein expression and β-DG protein expression in DG-KO mice and in reduced glycosylation of α-DG in α-DGN-KO mice (*SI Appendix*, Fig. S4). Notably, both DG-KO mice and α-DGN-KO mice exhibited higher PR8 viral titers in their lungs than their wild-type littermates on day 4 after infection [*P* = 0.0047 and *P* = 0.0079, Fig. 2 (relative data), *SI Appendix*, Fig. S5 (absolute data)]. These data demonstrate that α-DGN is required to optimally control viral load and suggest it may play an antiviral role during IAV infection.

### Overexpression of α-DGN or Treatment with Recombinant α-DGN Reduces Viral Titers in the Lungs after IAV Infection.

**Fig. 3.**
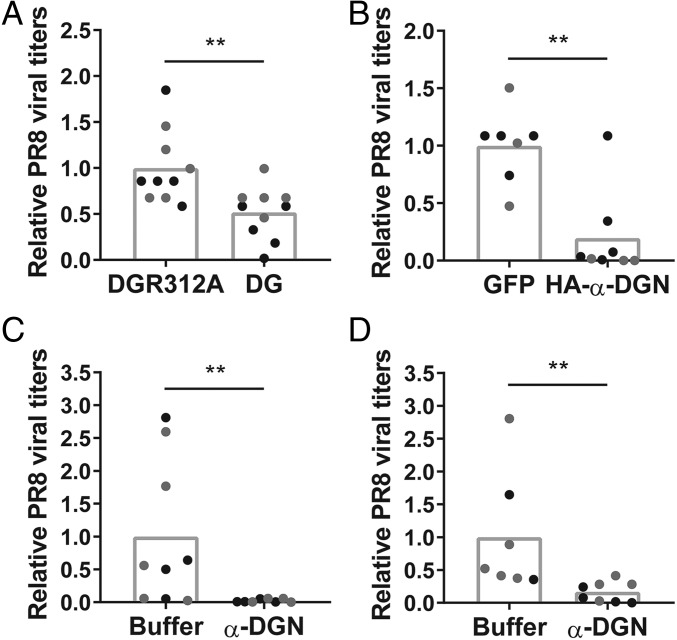
Overexpression of α-DGN or treatment with recombinant α-DGN reduces viral titers in the lungs after IAV infection. Relative titers of PR8 virus in whole lung tissue of C57BL/6 mice on day 4 after infection are shown. (*A*) Two weeks before infection, mice were infected with an adenovirus encoding wild-type DG (DG) or a mutated form of DG in which the N terminus cannot be cleaved (DGR312A). (*B*) Two weeks before infection, mice were infected with a GFP-expressing adenovirus or an HA-tagged α-DGN-GFP adenovirus. (*C*) Mice were treated with the elution buffer imidazole or recombinant His-tagged α-DGN 1 d before infection and on days 1 and 3 after infection. (*D*) Mice were treated with the elution buffer imidazole or recombinant His-tagged α-DGN on day 1, 2, and 3 after infection. Data are representative of two independent experiments with five mice (*A*), three to five mice (*B*), four to five mice (*C*), or two to five mice (*D*) per group in each experiment. Each dot represents an individual mouse: black dots represent mice in experiment 1; gray dots represent mice in experiment 2. Statistical analysis was performed using a Mann–Whitney *U* test. **P < 0.01.

Since the ablation of α-DGN increased the viral burden of PR8-infected mice, we next determined whether overexpression of α-DGN could enhance control of the virus during IAV infection. First, C57BL/6 mice were treated intranasally with a DG-expressing adenovirus or an adenovirus that encodes DG with a mutation in the furin recognition site of α-DG, preventing the cleavage of α-DGN (DGR312A). We predicted that after adenovirus infection, the amount of α-DGN in the BAL fluid of C57BL/6 mice infected with the DGR312A adenovirus would be lower than the amount of α-DGN in the BAL fluid of C57BL/6 mice infected with the DG adenovirus. Two weeks after intranasal administration of the adenoviruses, the levels of α-DGN in the BAL fluid were indeed lower in mice infected with the DGR312A adenovirus (*SI Appendix*, Fig. S6*A*). To determine if increased α-DGN in the BAL fluid had an impact on the response to the viral infection, we infected a larger set of C57BL/6 mice with these two adenoviruses 2 wk before PR8 infection and found that on day 4 after PR8 infection, PR8 viral titers in whole lung tissue of C57BL/6 mice that received the DG adenovirus were significantly lower relative to mice that received the DGR312A adenovirus [*P* = 0.0039, Fig. 3*A* (relative data), *SI Appendix*, Fig. S6*B* (absolute data)]. Thus, this suggests that elevated levels of α-DGN in the BAL fluid reduced viral titers in mice infected with PR8.

In a second experiment, we compared viral titers in whole lung tissue of PR8-infected C57BL/6 mice that received a control GFP-expressing adenovirus with those that received an HA-tagged α-DGN-GFP adenovirus and, thus, overexpressed α-DGN in the BAL fluid. Two weeks after administration of the adenoviruses, we confirmed the overexpression of α-DGN in the BAL fluid of a small set of C57BL/6 mice (*SI Appendix*, Fig. S7*A*). We next determined the effect of overexpressing α-DGN in the BAL fluid on the viral titers of mice infected with PR8. Mice overexpressing α-DGN in the BAL fluid had a significant decrease in PR8 viral titers in whole lung tissue relative to those that received the control adenovirus [*P* = 0.0037, Fig. 3*B* (relative data), *SI Appendix*, Fig. S7*B* (absolute data)], further supporting the role of α-DGN in regulating the response to IAV infection.

To further confirm that overexpression of α-DGN in the BAL fluid decreases viral titers in whole lung tissue of PR8-infected mice, we also treated PR8-infected C57BL/6 mice with recombinant His-tagged α-DGN protein. C57BL/6 mice received prophylactic intranasal treatment with His-tagged α-DGN, or received the elution buffer imidazole, 1 d before PR8 infection and received additional treatments on days 1 and 3 after infection. Four days after PR8 infection, significantly lower PR8 viral titers were observed in whole lung tissue of mice treated with His-tagged α-DGN than were found in mice that received the elution buffer imidazole [*P* = 0.0028, Fig. 3*C* (relative data), *SI Appendix*, Fig. S8*A* (absolute data)]. As prophylactic treatment of IAV is an unlikely clinical scenario, we next assessed PR8 viral titers in whole lung tissue of mice that received treatment with recombinant His-tagged α-DGN starting 1 d after PR8 infection and continuing on days 2 and 3 after infection. On day 4 after PR8 infection, significantly lower PR8 viral titers were observed in whole lung tissue of mice that received intranasal treatment with His-tagged α-DGN relative to mice that received the elution buffer imidazole [*P* = 0.0017, Fig. 3*D* (relative data), *SI Appendix*, Fig. S8*B* (absolute data)]. Collectively, these in vivo experiments suggest that the increased levels of α-DGN in the BAL fluid reduce the severity of IAV infection.

### α-DGN Reduces Influenza-Mediated Hemagglutination.

**Fig. 4.**
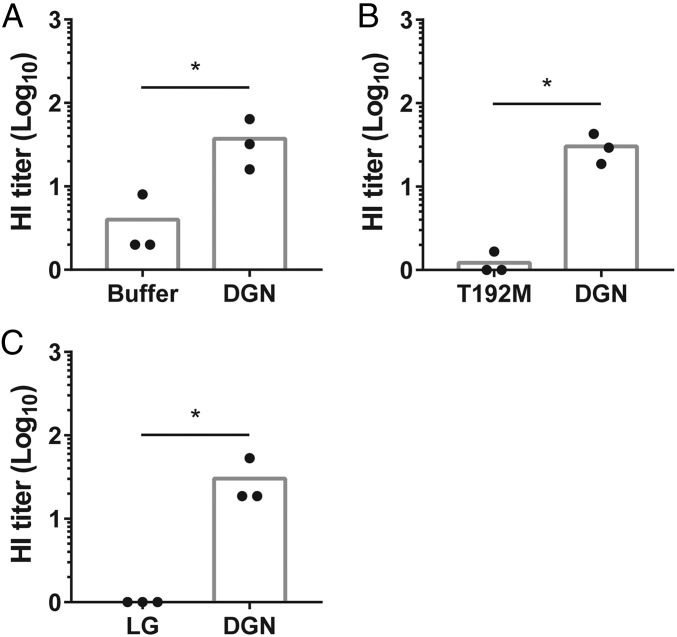
α-DGN reduces influenza-mediated hemagglutination. HI titers in the presence of recombinant His-tagged α-DGN (DGN) expressed by HEK293 cells compared with the elution buffer imidazole (Buffer) (*A*), recombinant α-DGN (DGN) produced by *E. coli* compared with the laminin G-4,5 domains of laminin-α1 (LG) produced by *E. coli* (*B*), or recombinant α-DGN (DGN) produced by *E. coli* compared with the mutant α-DGN-T192M *E. coli* protein (T192M) (*C*). Average HI titers are shown for three independent experiments with each experiment consisting of three technical replicates. Statistical analysis was performed using a Mann–Whitney *U* test. *P < 0.05.

Given the efficacy of reducing viral titers upon overexpression of α-DGN, we set out to determine the mechanisms by which α-DGN mediates its antiviral role during acute IAV infection. We hypothesized that α-DGN may reduce viral titers by neutralizing IAV. To test this hypothesis, we performed a series of hemagglutination inhibition (HI) assays (36), which determine the ability of red blood cells to bind to the hemagglutinin glycoprotein, the viral attachment protein that initiates cellular infection and is on the surface of the influenza virus particle. In the presence of a neutralizing agent, red blood cells and the influenza virus do not agglutinate as efficiently. Using this HI assay, we compared the ability of recombinant His-tagged α-DGN (expressed in and purified from HEK293 cells) to disrupt agglutination of red blood cells and the PR8 virus relative to the elution buffer imidazole and found a significant increase in the HI titers when His-tagged α-DGN was added (*P* = 0.0383, Fig. 4*A*), indicating that α-DGN disrupted agglutination.

To address the specificity of this interaction, we overexpressed α-DGN in *Escherichia coli* and purified it and an *E. coli*-derived control protein (the laminin G 4,5-domains of laminin-α1), and used both purified proteins in our HI assay. We again observed an increase in the HI titers in the presence of α-DGN, whereas this was not observed for the control protein (*P* = 0.0318, Fig. 4*B*), indicating that α-DGN can specifically neutralize IAV. We also compared *E. coli*-derived α-DGN protein with a mutated α-DGN-T192M protein expressed in and purified from *E. coli*. This mutant protein interrupts the interaction of α-DGN with LARGE1, resulting in reduced glycosylation of α-DG, thus disturbing its function as an extracellular matrix protein (37). Crystallographic analysis showed that the α-DGN region of the α-DGN-T190M protein (T192M mutation in human and rabbit; T190M mutation in mouse) adopts a conformational change and undergoes reorganization that potentially affects protein folding (38). In addition, small-angle X-ray scattering analysis showed that the T190M mutant, and also two other pathological point mutations in the N-terminal region of α-DG (V72I and D109N), altered the structural flexibility of α-DGN, which may explain why the interaction with LARGE1, and potentially other DG-modifying enzymes, is disturbed (39). In our HI assay, HI titers were significantly increased with the addition of the nonmutated α-DGN protein relative to the α-DGN-T192M protein (*P* = 0.0383, Fig. 4*C*), further indicating the specificity of α-DGN and suggesting its confirmation is also important in neutralizing IAV. These results demonstrate that α-DGN can neutralize IAV in vitro and suggest this may contribute to the antiviral effect of α-DGN observed in vivo.

## Discussion

Due to the involvement of α-DG in the dystroglycanopathies, most of the research on this protein is focused on its role in skeletal muscle. The dystroglycanopathies are a group of disorders that are caused by mutations in genes associated with the O-glycosylation of α-DG and include various forms of congenital muscular dystrophy and limb-girdle muscular dystrophy (40). Thus far, research in skeletal muscle has shown that α-DGN is necessary for the interaction of α-DG with the glycosyltransferase LARGE1, which initiates O-glycosylation of α-DG (17, 18). While research on α-DG in the lungs is limited, it is expressed in both the smooth muscle cells and the epithelial cells in the lungs (41). Furthermore, α-DG is involved in airway epithelial cell repair (42), epithelial morphogenesis of the lungs (43), and as a mechanoreceptor that transmits mechanical stretch forces by activating ERK1/2 and AMPK signaling cascades during normal respiration (44, 45). However, none of these studies have determined if α-DGN is secreted in the lungs or if it alters responses to IAV infection. In this study, we set out to determine whether inflammation induces *furin* expression and α-DGN secretion in the lungs. We show that IAV infection, which causes inflammation in the airways, indeed increases *furin* expression levels in whole lung tissue and that secreted α-DGN is detectable in murine BAL fluid. In addition, we show in vivo that absence of α-DGN significantly increases viral titers, while overexpressing α-DGN significantly reduces viral load after IAV infection. Finally, we show in vitro that α-DGN can neutralize IAV.

α-DGN has a high degree of sequence homology with the Ig kappa family (46) and may be increased during infections such as Lyme neuroborreliosis (24), suggesting it may have a protective function in the context of some diseases. The mouse-adapted IAV strain PR8 causes a localized infection in the lungs, limiting inflammation to the airways. As a result, this infection served as a useful model to study secreted α-DGN in the lungs in the context of inflammation, using a variety of tools (conditional knockout mice, overexpression studies, therapeutic applications). We chose to use IAV infection to induce inflammation, in addition to the proinflammatory stimulus CpG ODN (32), as this also gave us the opportunity to determine whether secreted α-DGN might have a protective function. The proprotein convertase furin can naturally cleave α-DGN from α-DG, thus reducing its ability to be O-glycosylated. Although we observed an increase in *furin* expression levels and reduced levels of α-DG glycosylation in the lungs of C57BL/6 mice after PR8 infection, we could not confirm that inflammation increased secreted α-DGN levels. We also found reduced levels of α-DG glycosylation in the lungs of C57BL/6 mice after intranasal CpG ODN treatment. As we observed reduced levels of α-DG glycosylation in the lungs either by IAV infection or by CpG ODN treatment, we believe that cleavage of α-DGN from α-DG is increased in both situations, supporting our hypothesis that inflammation increases *furin* expression levels leading to increased secretion of α-DGN. The decrease in α-DGN levels in the BAL fluid of PR8-infected C57BL/6 mice we observed following infection, as opposed to the expected increase in α-DGN levels, suggests that secreted α-DGN may have already been cleared from the BAL fluid or that α-DGN is retained within the cytosol while engaged in an, as of yet, unknown process and supports a potential antiviral function for α-DGN during IAV infection.

In line with this, we found that mice lacking DG or α-DGN had increased viral titers in whole lung tissue after PR8 infection, while overexpression of α-DGN decreased viral titers. Further, our HI assays suggested that α-DGN neutralizes IAV, as both recombinant His-α-DGN and *E. coli*-derived α-DGN reduced the binding of red blood cells to the hemagglutinin glycoprotein on the influenza virus particle. In human (HeLa) and mouse (C2C12) cells, and in human cerebrospinal fluid, α-DGN is a sialylated glycoprotein with both N- and O-linked glycans (22, 23). The fact that *E. coli*-derived α-DGN, which lacks glycans, also reduced influenza-mediated hemagglutination suggests that the presence of glycans on α-DGN does not play a major role in its antiviral effect. Future studies involving virus overlay protein binding assays and/or solid phase binding assays will be needed to determine if there is a direct interaction between α-DGN and PR8. In line with this, as α-DGN is a known interaction partner of LARGE1, we would like to perform future studies to establish potential structural similarities between LARGE1 and the IAV viral proteins. Alternatively, α-DGN may be competing with PR8 for receptors that are present on lung epithelial cells. To cause an infection, the hemagglutinin proteins on IAV bind to host cell receptors on lung epithelial cells that contain terminal α-2,6–linked or α-2,3–linked sialic acid moieties (47). As IAV is not the only virus that uses sialylated receptors in the lungs for infection (48), α-DGN may also play a protective function during other respiratory infections.

As muscle complications like pain and weakness are common during influenza infection in humans, a recent study looked at the effects of IAV infection in the context of muscular dystrophy using a zebrafish model of DMD. Using the same IAV strain that was used in our experiments, this study showed that systemic IAV infection leads to skeletal muscle fiber damage and increased inflammation in zebrafish skeletal muscle tissue, and that the extent of skeletal muscle fiber damage and mortality at early time points is exacerbated in zebrafish infected with PR8 (49). In our studies, we did not look at muscle pathology of the DG-KO mice and α-DGN-KO mice, although reduced α-DG (glycosylation) levels were observed in the skeletal muscles of these mice 2 mo after tamoxifen exposure. However, a previous study from our laboratory showed no overt necrosis and no difference in the percentage of centrally nucleated fibers and fiber size variation in DG-KO mice 3 mo after tamoxifen treatment. Also, at 2 mo after tamoxifen exposure, the specific forces in extensor digitorum longus muscles of DG-KO mice were comparable to those measured in control mice (34). Finally, we used intranasal delivery of the influenza virus, and not systemic delivery, which induces a localized respiratory infection in the lungs. Taken together, it seems unlikely that the increased PR8 viral titers that we observed in whole lung tissue of PR8-infected DG-KO mice and α-DGN-KO mice 2 mo after tamoxifen administration are due to muscle pathology of the respiratory muscles of these mice.

In this study, we show that *furin* expression levels in whole lung tissue are increased after IAV infection. Notably, furin inhibition is regarded a promising strategy for the short-term treatment of acute IAV infections (50), as furin activates various viral glycoproteins, including the hemagglutinin glycoprotein of several H5 and H7 influenza A strains (51). However, most influenza viruses, including the PR8 strain (H1N1 influenza A strain) that we used for our studies, do not contain a multibasic site (R-X-K/R-R motif) that is recognized by furin, and instead only contain a single arginine (monobasic site) that can, among others, be cleaved by trypsin-like serine proteases (52). Further, as inhibiting furin may affect the cleavage of α-DGN from α-DG, we believe that furin inhibition may only be a successful therapeutic strategy for a limited number of IAV infections.

We show in this study that overexpression of α-DGN in the lungs significantly reduced viral load after IAV infection, suggesting a protective role for the N-terminal fragment of α-DG in IAV proliferation with potential implications for its use as a treatment for IAV infection. All PR8 viral titers obtained in this study were determined on day 4 after infection, which, from our experience, is the day that the highest viral titers in whole lung tissue can be measured (35). However, disease burden on body weight is limited on day 4 after PR8 infection as mice typically start losing body weight at day 3 after infection, with the greatest severity in weight loss observed around day 8 after infection (35). In conclusion, to further determine whether overexpression of α-DGN in the lungs is a therapeutic option for IAV infection, studies in which mice are followed for a prolonged period of time after PR8 infection to establish disease burden on body weight and overall survival are needed.

## Materials and Methods

For details of mice, antibodies, and analysis, see *SI Appendix*, *SI Materials and Methods*.

### Antibodies.

Polyclonal DGN antibody Sheep173 was made by immunizing a sheep with a His-tagged rabbit DGN protein (amino acid 1–315 of α-DG) grown in a stable mammalian cell line [HEK293 cells; American Type Culture Collection (ATCC)] and purified by Talon beads (Takara Bio USA, Inc.). The anti-DGN antibodies in the sheep serum were affinity purified by affinity strips containing rabbit DGN purified from *E. coli* and transferred to a polyvinylidene difluoride membrane (Immobilon FL-Membrane; Millipore) to enrich antibodies to α-DGN lacking glycans and, thus, exclude any carbohydrate-specific antibody epitopes. Polyclonal α-DG antibody Sheep174 targeting the mucin region of α-DG (amino acid 316–485 of α-DG) was made by immunizing a sheep with a His-tagged protein grown in a stable mammalian cell line (HEK293 cells; ATCC) and purified by Talon beads (Takara Bio USA, Inc.). The anti-α-DG antibodies to the mucin region were affinity purified by affinity strips containing GST-mucin α-DG purified from *E. coli* and transferred to a polyvinylidene difluoride membrane (Immobilon FL-Membrane; Millipore). Using *E. coli* protein of the mucin α-DG region for affinity purification ensured we enriched for antibodies to α-DG lacking glycans in the mucin region and excluded any carbohydrate-specific antibody epitopes.

## Supplementary Material

Supplementary File

## References

[1] Ervasti J. M., Campbell K. P., Membrane organization of the dystrophin-glycoprotein complex. Cell 66, 1121–1131 (1991). 1913804 10.1016/0092-8674(91)90035-w

[2] Ibraghimov-Beskrovnaya O., Primary structure of dystrophin-associated glycoproteins linking dystrophin to the extracellular matrix. Nature 355, 696–702 (1992). 1741056 10.1038/355696a0

[3] Ervasti J. M., Campbell K. P., A role for the dystrophin-glycoprotein complex as a transmembrane linker between laminin and actin. J. Cell Biol. 122, 809–823 (1993). 8349731 10.1083/jcb.122.4.809 PMC2119587

[4] Durbeej M., Henry M. D., Ferletta M., Campbell K. P., Ekblom P., Distribution of dystroglycan in normal adult mouse tissues. J. Histochem. Cytochem. 46, 449–457 (1998). 9524190 10.1177/002215549804600404

[5] Henry M. D., Campbell K. P., A role for dystroglycan in basement membrane assembly. Cell 95, 859–870 (1998). 9865703 10.1016/s0092-8674(00)81708-0

[6] Cohn R. D., Disruption of DAG1 in differentiated skeletal muscle reveals a role for dystroglycan in muscle regeneration. Cell 110, 639–648 (2002). 12230980 10.1016/s0092-8674(02)00907-8

[7] Cao W., Identification of alpha-dystroglycan as a receptor for lymphocytic choriomeningitis virus and Lassa fever virus. Science 282, 2079–2081 (1998). 9851928 10.1126/science.282.5396.2079

[8] Akhavan A., Crivelli S. N., Singh M., Lingappa V. R., Muschler J. L., SEA domain proteolysis determines the functional composition of dystroglycan. FASEB J. 22, 612–621 (2008). 17905726 10.1096/fj.07-8354com

[9] Suzuki A., Molecular organization at the glycoprotein-complex-binding site of dystrophin. Three dystrophin-associated proteins bind directly to the carboxy-terminal portion of dystrophin. Eur. J. Biochem. 220, 283–292 (1994). 8125086 10.1111/j.1432-1033.1994.tb18624.x

[10] Jung D., Yang B., Meyer J., Chamberlain J. S., Campbell K. P., Identification and characterization of the dystrophin anchoring site on beta-dystroglycan. J. Biol. Chem. 270, 27305–27310 (1995). 7592992 10.1074/jbc.270.45.27305

[11] Gee S. H., Montanaro F., Lindenbaum M. H., Carbonetto S., Dystroglycan-alpha, a dystrophin-associated glycoprotein, is a functional agrin receptor. Cell 77, 675–686 (1994). 8205617 10.1016/0092-8674(94)90052-3

[12] Peng H. B., The relationship between perlecan and dystroglycan and its implication in the formation of the neuromuscular junction. Cell Adhes. Commun. 5, 475–489 (1998). 9791728 10.3109/15419069809005605

[13] Sugita S., A stoichiometric complex of neurexins and dystroglycan in brain. J. Cell Biol. 154, 435–445 (2001). 11470830 10.1083/jcb.200105003 PMC2150755

[14] Sato S., Pikachurin, a dystroglycan ligand, is essential for photoreceptor ribbon synapse formation. Nat. Neurosci. 11, 923–931 (2008). 18641643 10.1038/nn.2160

[15] Chiba A., Structures of sialylated O-linked oligosaccharides of bovine peripheral nerve alpha-dystroglycan. The role of a novel O-mannosyl-type oligosaccharide in the binding of alpha-dystroglycan with laminin. J. Biol. Chem. 272, 2156–2162 (1997). 8999917 10.1074/jbc.272.4.2156

[16] Michele D. E., Post-translational disruption of dystroglycan-ligand interactions in congenital muscular dystrophies. Nature 418, 417–422 (2002). 12140558 10.1038/nature00837

[17] Kanagawa M., Molecular recognition by LARGE is essential for expression of functional dystroglycan. Cell 117, 953–964 (2004). 15210115 10.1016/j.cell.2004.06.003

[18] Yoshida-Moriguchi T., O-mannosyl phosphorylation of alpha-dystroglycan is required for laminin binding. Science 327, 88–92 (2010). 20044576 10.1126/science.1180512 PMC2978000

[19] Singh J., Proteolytic enzymes and altered glycosylation modulate dystroglycan function in carcinoma cells. Cancer Res. 64, 6152–6159 (2004). 15342399 10.1158/0008-5472.CAN-04-1638

[20] Brancaccio A., Schulthess T., Gesemann M., Engel J., The N-terminal region of alpha-dystroglycan is an autonomous globular domain. Eur. J. Biochem. 246, 166–172 (1997). 9210479 10.1111/j.1432-1033.1997.00166.x

[21] Bozic D., Sciandra F., Lamba D., Brancaccio A., The structure of the N-terminal region of murine skeletal muscle alpha-dystroglycan discloses a modular architecture. J. Biol. Chem. 279, 44812–44816 (2004). 15326183 10.1074/jbc.C400353200

[22] Saito F., Saito-Arai Y., Nakamura A., Shimizu T., Matsumura K., Processing and secretion of the N-terminal domain of alpha-dystroglycan in cell culture media. FEBS Lett. 582, 439–444 (2008). 18201566 10.1016/j.febslet.2008.01.006

[23] Saito F., Secretion of N-terminal domain of α-dystroglycan in cerebrospinal fluid. Biochem. Biophys. Res. Commun. 411, 365–369 (2011). 21741360 10.1016/j.bbrc.2011.06.150

[24] Hesse C., The N-terminal domain of α-dystroglycan, released as a 38 kDa protein, is increased in cerebrospinal fluid in patients with Lyme neuroborreliosis. Biochem. Biophys. Res. Commun. 412, 494–499 (2011). 21843510 10.1016/j.bbrc.2011.07.129

[25] Hall H., Bozic D., Michel K., Hubbell J. A., N-terminal alpha-dystroglycan binds to different extracellular matrix molecules expressed in regenerating peripheral nerves in a protein-mediated manner and promotes neurite extension of PC12 cells. Mol. Cell. Neurosci. 24, 1062–1073 (2003). 14697669 10.1016/j.mcn.2003.08.007

[26] Heng S., Evans J., Salamonsen L. A., Jobling T. W., Nie G., The significance of post-translational removal of α-DG-N in early stage endometrial cancer development. Oncotarget 8, 81942–81952 (2017). 29137235 10.18632/oncotarget.17286 PMC5669861

[27] Crowe K. E., Shao G., Flanigan K. M., Martin P. T., N-terminal α dystroglycan (αDG-N): A Potential serum biomarker for Duchenne muscular dystrophy. J. Neuromuscul. Dis. 3, 247–260 (2016). 27854211 10.3233/JND-150127 PMC5541672

[28] Jaaks P., Bernasconi M., The proprotein convertase furin in tumour progression. Int. J. Cancer 141, 654–663 (2017). 28369813 10.1002/ijc.30714

[29] Turpeinen H., Proprotein convertases in human atherosclerotic plaques: The overexpression of FURIN and its substrate cytokines BAFF and APRIL. Atherosclerosis 219, 799–806 (2011). 21889147 10.1016/j.atherosclerosis.2011.08.011

[30] Pesu M., Muul L., Kanno Y., O’Shea J. J., Proprotein convertase furin is preferentially expressed in T helper 1 cells and regulates interferon gamma. Blood 108, 983–985 (2006). 16627761 10.1182/blood-2005-09-3824 PMC1895858

[31] Lin H., Protective role of systemic furin in immune response-induced arthritis. Arthritis Rheum. 64, 2878–2886 (2012). 22605541 10.1002/art.34523

[32] Knuefermann P., CpG oligonucleotide activates Toll-like receptor 9 and causes lung inflammation in vivo. Respir. Res. 8, 72 (2007). 17925007 10.1186/1465-9921-8-72 PMC2173891

[33] Williamson R. A., Dystroglycan is essential for early embryonic development: Disruption of Reichert’s membrane in dag1-null mice. Hum. Mol. Genet. 6, 831–841 (1997). 9175728 10.1093/hmg/6.6.831

[34] Rader E. P., Role of dystroglycan in limiting contraction-induced injury to the sarcomeric cytoskeleton of mature skeletal muscle. Proc. Natl. Acad. Sci. U.S.A. 113, 10992–10997 (2016). 27625424 10.1073/pnas.1605265113 PMC5047148

[35] Slütter B., Pewe L. L., Lauer P., Harty J. T., Cutting edge: Rapid boosting of cross-reactive memory CD8 T cells broadens the protective capacity of the Flumist vaccine. J. Immunol. 190, 3854–3858 (2013). 23467935 10.4049/jimmunol.1202790 PMC3622175

[36] Hirst G. K., The quantitative determination of influenza virus and antibodies by means of red cell agglutination. J. Exp. Med. 75, 49–64 (1942). 19871167 10.1084/jem.75.1.49 PMC2135212

[37] Hara Y., A dystroglycan mutation associated with limb-girdle muscular dystrophy. N. Engl. J. Med. 364, 939–946 (2011). 21388311 10.1056/NEJMoa1006939 PMC3071687

[38] Bozzi M., The structure of the T190M mutant of murine α-Dystroglycan at high resolution: Insight into the molecular basis of a primary dystroglycanopathy. PLoS One 10, e0124277 (2015). 25932631 10.1371/journal.pone.0124277 PMC4416926

[39] Covaceuszach S., The effect of the pathological V72I, D109N and T190M missense mutations on the molecular structure of α-dystroglycan. PLoS One 12, e0186110 (2017). 29036200 10.1371/journal.pone.0186110 PMC5643065

[40] Taniguchi-Ikeda M., Morioka I., Iijima K., Toda T., Mechanistic aspects of the formation of α-dystroglycan and therapeutic research for the treatment of α-dystroglycanopathy: A review. Mol. Aspects Med. 51, 115–124 (2016). 27421908 10.1016/j.mam.2016.07.003

[41] Durbeej M., Campbell K. P., Biochemical characterization of the epithelial dystroglycan complex. J. Biol. Chem. 274, 26609–26616 (1999). 10473626 10.1074/jbc.274.37.26609

[42] White S. R., Wojcik K. R., Gruenert D., Sun S., Dorscheid D. R., Airway epithelial cell wound repair mediated by alpha-dystroglycan. Am. J. Respir. Cell Mol. Biol. 24, 179–186 (2001). 11159052 10.1165/ajrcmb.24.2.3993

[43] Durbeej M., Dystroglycan binding to laminin alpha1LG4 module influences epithelial morphogenesis of salivary gland and lung in vitro. Differentiation 69, 121–134 (2001). 11798066 10.1046/j.1432-0436.2001.690206.x

[44] Budinger G. R., Stretch-induced activation of AMP kinase in the lung requires dystroglycan. Am. J. Respir. Cell Mol. Biol. 39, 666–672 (2008). 18556591 10.1165/rcmb.2007-0432OC PMC2586043

[45] Takawira D., Budinger G. R., Hopkinson S. B., Jones J. C., A dystroglycan/plectin scaffold mediates mechanical pathway bifurcation in lung epithelial cells. J. Biol. Chem. 286, 6301–6310 (2011). 21149456 10.1074/jbc.M110.178988 PMC3057847

[46] Bozic D., Engel J., Brancaccio A., Sequence analysis suggests the presence of an IG-like domain in the N-terminal region of alpha-dystroglycan which was crystallized after mutation of a protease susceptible site (Arg168–>His). Matrix Biol. 17, 495–500 (1998). 9881601 10.1016/s0945-053x(98)90097-x

[47] Medina R. A., García-Sastre A., Influenza a viruses: New research developments. Nat. Rev. Microbiol. 9, 590–603 (2011). 21747392 10.1038/nrmicro2613 PMC10433403

[48] Matrosovich M., Herrler G., Klenk H. D., Sialic acid receptors of viruses. Top. Curr. Chem. 367, 1–28 (2015). 23873408 10.1007/128_2013_466 PMC7120183

[49] Goody M., Jurczyszak D., Kim C., Henry C., Influenza a virus infection damages zebrafish skeletal muscle and exacerbates disease in zebrafish modeling Duchenne muscular dystrophy. PLoS Curr. 9, ecurrents.md.8a7e35c50fa2b48156799d3c39788175 (2017). 29188128 10.1371/currents.md.8a7e35c50fa2b48156799d3c39788175 PMC5693338

[50] Hardes K., Novel furin inhibitors with potent anti-infectious activity. ChemMedChem 10, 1218–1231 (2015). 25974265 10.1002/cmdc.201500103

[51] Stieneke-Gröber A., Influenza virus hemagglutinin with multibasic cleavage site is activated by furin, a subtilisin-like endoprotease. EMBO J. 11, 2407–2414 (1992). 1628614 10.1002/j.1460-2075.1992.tb05305.x PMC556715

[52] Bahgat M. M., Błazejewska P., Schughart K., Inhibition of lung serine proteases in mice: A potentially new approach to control influenza infection. Virol. J. 8, 27 (2011). 21251300 10.1186/1743-422X-8-27 PMC3034701

