# Supplementary material for: Protective role for the N-terminal domain of α-dystroglycan in Influenza A virus proliferation
Source: Proc Natl Acad Sci U S A. 2019 May 16;116(23):11396–401. doi: 10.1073/pnas.1904493116 (PMC6561248; doi:10.1073/pnas.1904493116)

Supplementary Information for:

A protective role for the N-terminal domain of α-dystroglycan in Influenza A virus proliferation

Jessica C. de Greef[1,2,3#], Bram Slütter[4&], Mary E. Anderson[1,2,3], Rebecca Hamlyn[1,2,3], Raul O'Campo Landa[1,2,3], Ellison J. McNutt[1,2,3], Yuji Hara[1,2,3], Lecia L. Pewe[4], David Venzke[1,2,3], Kiichiro Matsumura[5], Fumiaki Saito[5], John T. Harty[4,6], Kevin P. Campbell[1,2,3*]

[1]Howard Hughes Medical Institute; [2]Department of Molecular Physiology and Biophysics, The University of Iowa, Iowa City, IA 52242; [3]Department of Neurology, The University of Iowa, Iowa City, IA 52242; [4]Department of Microbiology and Immunology, The University of Iowa, Iowa City, IA 52242; [5]Department of Neurology, Teikyo University School of Medicine, Itabashi-ku, Tokyo 173-8605, Japan; [6]Department of Pathology, The University of Iowa, Iowa City, IA 52242.

[*]Correspondence: kevin-campbell@uiowa.edu.

www.pnas.org/cgi/doi/10.1073/pnas.1904493116

Supplementary Materials and Methods

Animals

Animal care, ethical use, and procedures were approved by the National Institutes of Health and the Animal Care Use and Review Committee at the University of Iowa, and were performed in strict accordance with these protocols. At the University of Iowa, all mice are housed socially (unless single housing is required), under specific pathogen-free conditions in an AAALAC accredited animal facility. Housing conditions are as specified in the Guide for the Care and Use of Laboratory Animals (National Research Council). Mice are housed on HEPA-filtered ventilated racks, in solid-bottom cages (Thoren Caging Systems Inc., Hazleton, PA) with mixed paper bedding. A standard 12h/12h light/dark cycle is used. Standard rodent chow (Harlan Laboratories, Indianapolis, IN) and water are available *ad libitum*. Mice homozygous for a floxed allele of DG, in which loxP sites flank exon 2 of *Dag1* (#009652; The Jackson Laboratory, Bar Harbor, ME), were crossed with transgenic mice expressing a tamoxifen-inducible Cre-mediated recombination system (#004682; The Jackson Laboratory, Bar Harbor, ME). Inducible DG-KO mice were Cre-positive and homozygous for floxed *Dag1*; control mice were homozygous for floxed *Dag1* but Cre-negative. Inducible α-DGN KO mice were generated by replacing exon 3 of *Dag1*, which contains the ATG initiation codon, with a construct containing DG cDNA lacking amino acids 31-314, a SV40polyA, and the loxP-Neo cassette (1). Inducible α-DGN-KO mice were obtained by crossing mice lacking α-DGN on one allele with Cre-positive mice that were heterozygous for the floxed *Dag1* allele. Inducible α-DGN-KO mice were Cre-positive, lacked α-DGN on one allele, and carried floxed *Dag1* on the second allele. Control mice were Cre-negative, lacked α-DGN on one allele, and carried floxed *Dag1* on the second allele. At 8 weeks of age, tamoxifen (200 mg/kg) was administered to all mice by oral gavage on two occasions within a one-week period. DG-KO mice and α-DGN KO mice were infected with PR8 two months later. C57Bl/6 mice were obtained from The Jackson Laboratory (#000664; The Jackson Laboratory, Bar Harbor, ME). At 8 weeks of age, C57Bl/6 mice received adenovirus by intranasal treatment. C57Bl/6 mice were infected with PR8 or received CpG ODN treatment at 10 weeks of age.

Antibodies

The following antibodies have been described previously and were obtained from the listed sources: IIH6 monoclonal antibody (Campbell laboratory, 2), monoclonal β-DG antibody 8D5 (Campbell laboratory, 3), and α-DG/β-DG antibody AF6868 (R&D Systems, Minneapolis, MN).

HEK293 cells stably expressing HA-tagged α-DGN or His-tagged α-DGN

HEK293 cells (ATCC, Manassas, VA) were maintained at 37°C and 5% CO_2 in Dulbecco's modified Eagle's medium (DMEM) supplemented with 10% fetal bovine serum, 2 mM glutamine, and 1% penicillin-streptomycin (all Gibco; Thermo Fisher Scientific, Waltham, MA). HEK293 cells were transfected with the pcDNA3.1 mammalian expression vector (Thermo Fisher Scientific, Waltham, MA) containing the complete N-terminal sequence of rabbit α-DGN with the His-tag or HA-tag inserted directly after the signal peptide sequence using FuGene 6 Transfection Reagent (Promega, Madison, WI). Cells stably expressing HA-tagged rabbit α-DGN or His-tagged rabbit α-DGN were cultured in DMEM supplemented with G418 (Thermo Fisher Scientific, Waltham, MA).

Generation of recombinant His-tagged α-DGN

HEK293 cells stably expressing His-tagged rabbit α-DGN were adapted to serum-free medium, 293SFMII (Thermo Fisher Scientific, Waltham, MA), and cultivated in CELLine bioreactors (CL1000; Argos Technologies, Vernon Hills, IL). His-tagged rabbit α-DGN secreted into the culture medium was purified using the Talon metal-affinity resin (Takara Bio USA Inc., Mountain View, CA) according to the manufacturer's instructions. The purity of the protein was confirmed by SDS-PAGE and Coomassie Brilliant Blue staining. 50 µl of purified His-tagged rabbit α-DGN (~5 µg/µl; concentration was batch-dependent, one batch was used for one *in vivo* experiment) was given intranasally to mice that were lightly anesthetized with isoflurane.

Production and purification of *E.coli* proteins

The recombinant wild-type rabbit α-DGN (amino acid 50-313) and the recombinant rabbit α-DGN T192M proteins were expressed as described previously with minor modifications (4). The expressed proteins were initially purified using a cobalt affinity column (Talon; Takara Bio USA Inc., Mountain View, CA). The purified proteins were then pooled and concentrated using Amicon Ultra-15 centrifugal filter units with a molecular weight cut-off of 10 kDa (Merck Millipore, Burlington, MA). After ion-exchange chromatography to remove imidazole from the buffer, the proteins were cut by thrombin, which released products of the thioredoxin fusion protein containing the 6xHis-tag and the DGN proteins. This digest was placed again on a cobalt affinity column (Talon; Takara Bio USA Inc., Mountain View, CA) with the flow through of the column containing the recombinant DGN proteins. The proteins were further purified using gel filtration chromatography as described (4). The plasmid used to produce the laminin-α1 protein containing the domains LG4 and LG5 contained a GST fusion protein as well as a myc tag on the C-terminus. The plasmid was transfected into *E.coli* BL21 (DE3) cells, and the cells were grown at 37°C to an OD600 of 0.6 in LB media. Next, the cells were induced with 0.1 mM IPTG, incubated at 25°C for 6 hours, harvested and lysed, and spun down at 10,000 x g using a JA-10 rotor (Beckman Coulter, Brea, CA). The supernatant containing the expressed protein was loaded onto a Glutathione Sepharose 4B column (GE Healthcare, Chicago, IL), washed with PBS, and eluted with 0.2 M Glutathione. Eluted fractions were analyzed by SDS-PAGE and pooled. Finally, samples were dialyzed into PBS.

RNA isolation, cDNA synthesis, and real-time quantitative PCR

Total RNA was isolated from mouse lungs using TRIzol (Thermo Fisher Scientific, Waltham, MA) according to the manufacturer's protocol, followed by purification with the RNeasy isolation kit (Qiagen, Valencia, CA). First-strand cDNA was synthesized from 2 µg of total RNA using the Omniscript reverse transcriptase (Qiagen, Valencia, CA) and oligo(dT)$_{18}$ primers (Thermo Fisher Scientific, Waltham, MA), according to the manufacturer's instructions. The furin gene was amplified from cDNA using quantitative real-time PCR and the following primers: 5'-ACAACTATGGGACGCTGACC-3' and 5'-

GGCTGGATGTGAGGGTCTT-3'. *Rpl4* and *Rps29* were used as normalization controls, and sequences are available upon request. cDNA levels were determined using SYBR green (MilliporeSigma, St. Louis, MO; 100,000-fold dilution) and fluorescein (BioRad, Hercules, CA; 100,000-fold dilution) in a MyiQ RT-PCR detection system (BioRad, Hercules, CA). All samples were run in triplicate.

Protein isolation, wheat germ agglutinin purification, and western blot analysis

Whole lung tissue and skeletal muscle were homogenized using a Brinkmann Polytron Homogenizer (PT 10-35; Thermo Fisher Scientific, Waltham, MA) in 10 volumes of solubilization buffer (150 mM NaCl, 50 mM Tris, 200 mM PMSF, 100 mM benzamidine, pH 7.4) with 1% Triton X-100. Subsequently, samples were incubated rotating top-over-top for 1 hour at 4°C and spun for 30 minutes at maximum speed at 4°C (tabletop centrifuge; Eppendorf, Hauppauge, NY) to remove non-homogenized material. Next, solubilized supernatants were incubated overnight with wheat germ agglutinin (WGA) agarose (Vector Laboratories, Burlingame, CA), followed by washing in solubilization buffer supplemented with 0.1% Triton X-100 and were subsequently eluted with 0.3 M N-acetylglucosamine. WGA-purified samples were separated by 3-15% SDS-PAGE and transferred to polyvinylidene difluoride membranes (Immobilon FL-Membrane; Millipore, Billerica, MA). The membranes were blocked in 5% milk or 2% fish gelatin (MilliporeSigma, St. Louis, MO) in Tris-buffered saline (TBS) containing 0.1% Tween-20 and incubated overnight with primary antibodies. Next, blots were washed with TBS containing 0.1% Tween-20 and incubated with dye-conjugated secondary antibodies (Rockland Immunochemicals Inc, Boyertown, PA). After washing, blots were imaged using the Odyssey Imaging System (LI-COR Biosciences, Lincoln, NE).

BAL fluid isolation

Bronchoalveolar lavage was performed post mortem by inflating the lungs with 1 ml of PBS through the trachea. This procedure was repeated 3 times using the same 1 ml of PBS.

Adenovirus infections

Mice were infected intranasally with $2.5x10^8$ plaque-forming units of E1-deficient recombinant adenovirus in 50 µl PBS, while lightly anesthetized with isoflurane. Mouse furin recombinant adenovirus (ADV-259702), containing a CMV promoter, was obtained from Vector Biolabs (Malvern, PA). The HA-tagged α-DGN-GFP recombinant adenovirus was custom made by Vector Biolabs (Malvern, PA); the cDNA construct contained amino acids 1-310 of mouse *Dag1* (NP_034147), with an HA tag between amino acid 27 and 28 (amino acids 1-27 encode the signal peptide), a CMV promoter, and GFP. The AdV-EGFP and AdV-DG recombinant adenoviruses have been described previously (5, 6) and were purified by the University of Iowa Gene Transfer Vector Core. The AdV-DGR312A recombinant adenovirus was generated using a α-DG-Fc fusion protein (DGFc5R312A, 53). The University of Iowa Gene Transfer Vector Core generated this recombinant adenovirus by standard methods as previously described (7).

PR8 infection

A/Puerto Rico/08/34 influenza virus was grown in chicken eggs as previously described (8). While lightly anesthetized with isoflurane, mice were infected intranasally with 10-fold LD50 (50% lethal dose) in 50 µl PBS (2×10^5 tissue culture infective dose (TCID50)).

CpG ODN treatment

While lightly anesthetized with isoflurane, mice were treated intranasally with CpG ODN 1826 (100 µg in 50 µl PBS; InvivoGen, San Diego, CA) on two separate days (three days apart). Mice were sacrificed one day after the final treatment.

α-DGN ELISA

High binding 96-wells plates (Costar; Thermo Fisher Scientific, Waltham, MA) were incubated overnight at 4ºC with BAL fluid (in triplicate) or medium containing secreted HA-tagged α-DGN (positive control; to generate a linear standard curve). The following day, after a washing step with TBS, the plates were first blocked for 2 hours at room temperature with 3% BSA/TBS. Next, the plates were incubated for 2 hours at

room temperature with the α-DGN antibody Sheep173 (1:500 dilution) or no antibody (negative control) in 3% BSA/TBS containing 0.1% Triton X-100, followed by four washing steps with 1% BSA/TBS containing 0.1% Triton X-100. Incubation with the secondary antibody anti-sheep-IgG-horseradish peroxidase was for 1 hour at room temperature in 1% BSA/TBS containing 0.1% Triton X-100. After an additional four washing steps with 1% BSA/TBS containing 0.1% Triton X-100, the plates were incubated with 3,3',5,5'-tetramethylbenzidine solution (Thermo Fisher Scientific, Waltham, MA) for 15 minutes. The reaction was stopped with 2 M H_2SO_4 and the plates were read at 450 nm with a plate reader (SpectraMax 190 Microplate Reader; Molecular Devices, San Jose, CA) to determine absorbance. α-DGN concentrations were obtained by subtracting the average OD450 signal obtained from negative wells stained with the secondary antibody only from the average OD450 signal obtained from wells stained with both the primary and the secondary antibody. The concentration was extrapolated from the standard curve obtained with medium-containing secreted HA-tagged α-DGN.

HI assay

Serial dilutions of α-DGN or control protein (start quantity of 1 µg) were incubated with $4x10^{10}$ TCID50 PR8 in PBS for 30 minutes. To assess hemagglutination, supernatant was mixed 1:1 with 0.5% v/v chicken red blood cells (Lampire Biological Lab Inc, Ottsville, PA) in PBS and incubated for 60 minutes at 4°C.

Viral titer determination

Whole lung tissue was homogenized in 2 ml DMEM (Gibco; Thermo Fisher Scientific, Waltham, MA). Serial dilutions of lung homogenates were co-seeded in 96 well plates with $1x10^5$ Madin-Darby canine kidney cells per well and incubated at 37°C and 5% CO_2 in DMEM medium. The next day, medium was replaced with DMEM containing 50 µg/ml gentamicin, penicillin, streptomycin, and 0.001% trypsin (all Gibco; Thermo Fisher Scientific, Waltham, MA) and incubated for an additional 72 hours. To assess hemagglutination, supernatant was mixed 1:1 with 0.5% v/v chicken red blood cells (Lampire Biological Lab Inc, Ottsville, PA) in PBS and incubated for 60 minutes at 4°C.

Statistical analysis

Statistical analysis was performed using GraphPad Prism software (version 7.00; GraphPad Software Inc., La Jolla, CA). For most comparisons, data were analyzed using the Student's *t*-test or the Mann-Whitney U test, depending on whether normality and equal variance could be assumed. Normality was determined by Shapiro-Wilk normality tests. For comparison of α-DGN levels in the BAL fluid of PR8-infected C57Bl/6 mice, data were analyzed using Kruskal-Wallis analysis of variance on ranks followed by Dunn's multiple comparison tests.

Supplementary References

1. Rader EP, et al. (2016). Role of dystroglycan in limiting contraction-induced injury to the sarcomeric cytoskeleton of mature skeletal muscle. *Proc Natl Acad Sci U S A* 113: 10992-10997.
2. Ervasti JM, Campbell KP. (1991). Membrane organization of the dystrophin-glycoprotein complex. *Cell* 66: 1121-1131.
3. Ibraghimov-Beskrovnaya O, et al. (1992). Primary structure of dystrophin-associated glycoproteins linking dystrophin to the extracellular matrix. *Nature* 355: 696-702.
4. Bozzi M, et al. (2015). The Structure of the T190M Mutant of Murine α-Dystroglycan at High Resolution: Insight into the Molecular Basis of a Primary Dystroglycanopathy. *PLoS One* 10: e0124277.
5. Kunz S, Sevilla N, McGavern DB, Campbell KP, Oldstone MB. (2001). Molecular analysis of the interaction of LCMV with its cellular receptor [alpha]-dystroglycan. *J Cell Biol* 155: 301-310.
6. Barresi R, et al. (2004). LARGE can functionally bypass alpha-dystroglycan glycosylation defects in distinct congenital muscular dystrophies. *Nat Med* 10: 696-703.
7. Duclos F, et al. (1998). Progressive muscular dystrophy in alpha-sarcoglycan-deficient mice. *J Cell Biol* 142: 1461-1471.
8. Legge KL, Braciale TJ. (2005). Lymph node dendritic cells control CD8+ T cell responses through regulated FasL expression. *Immunity* 23: 649-659.

Supplementary Figures

Fig. S1. Infection with IAV elevates *Furin* expression in the lungs.

Absolute data for *Furin* expression in whole lung tissue of PR8-infected C57Bl/6 mice 6 days post infection. Left graph shows experiment 1; right graph shows experiment 2. Each dot represents an individual mouse. *Furin* expression is relative to the housekeeping genes *Rpl4* and *Rps29*.

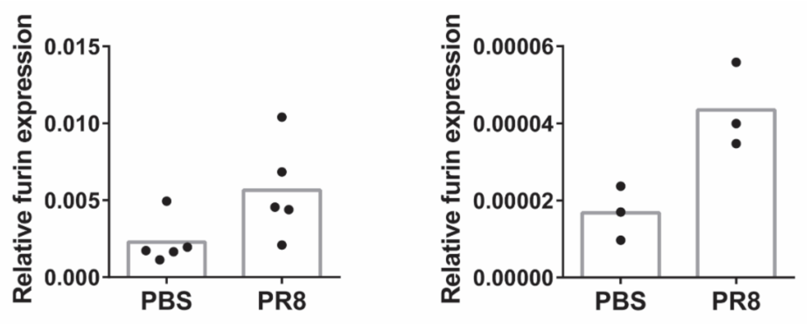

Fig. S2. Sheep173 is a novel polyclonal antibody that detects α-DGN protein.

α-DGN protein expression in medium obtained from regular HEK293 cultures and from HEK293 cultures that stably express HA-tagged α-DGN. The α-DGN antibody, Sheep173, is an affinity-purified sheep polyclonal antibody that was made using the complete N-terminal region of α-DG. In each lane, medium removed from HEK293 cells was loaded. Lane 1 and 2 contain medium obtained from regular HEK293 cells after 1 hour in culture (d0) and after 48 hours in culture (d2). Lane 3 and 4 contain medium obtained from HEK293 cells that are stably expressing HA-tagged α-DGN after 1 hour in culture (d0) and after 48 hours in culture (d2). Data are representative of 2 independent experiments. Protein size indicated is in kDa.

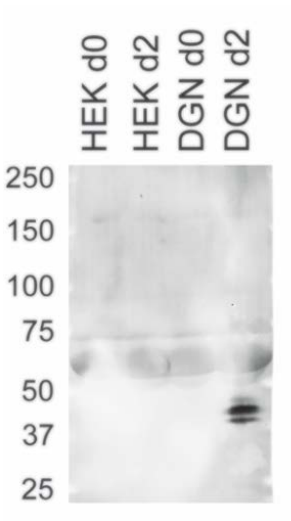

Fig. S3. Infection with IAV decreases α-DGN levels in the bronchoalveolar lavage fluid.

Absolute data for α-DGN levels in the BAL fluid of PR8-infected C57Bl/6 mice at the indicated day following infection. Left graph shows experiment 1; right graph shows experiment 2. Each dot represents an individual mouse.

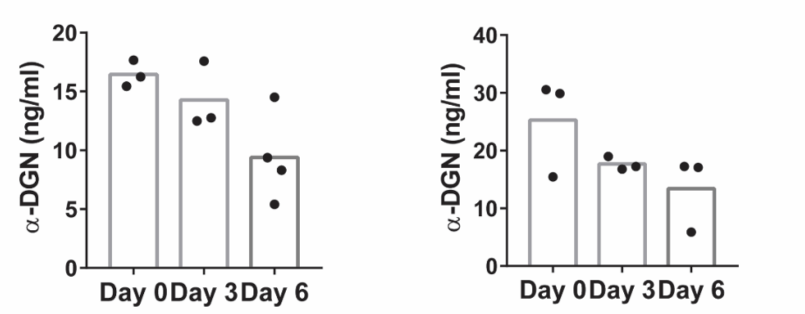

Fig. S4. α-DG protein expression, α-DG glycosylation levels, and β-DG protein expression in skeletal muscle of DG-KO mice and α-DGN-KO mice following administration of tamoxifen.

A. α-DG protein expression and β-DG protein expression (left) and α-DG glycosylation levels (right) in skeletal muscle of control mice (WT) and those that lack DG (DG-KO) two months after administration of tamoxifen. Data are representative of 2 independent experiments. Each lane represents a distinct mouse; protein size indicated is in kDa. The AF6868 antibody detects both α-DG (it binds to α-DG even in the absence of glycosylation) and β-DG; the IIH6 antibody detects the glycosylated form of α-DG (*).

B. α-DG protein expression and β-DG protein expression (left) and α-DG glycosylation levels (right) in skeletal muscle of control mice (WT) and those that lack α-DGN (α-DGN-KO) two months after administration of tamoxifen. Data are representative of 2 independent experiments. Each lane represents a distinct mouse; protein size indicated is in kDa. The AF6868 antibody detects both α-DG (it binds to α-DG even in the absence of glycosylation) and β-DG; the IIH6 antibody detects the glycosylated form of α-DG (*).

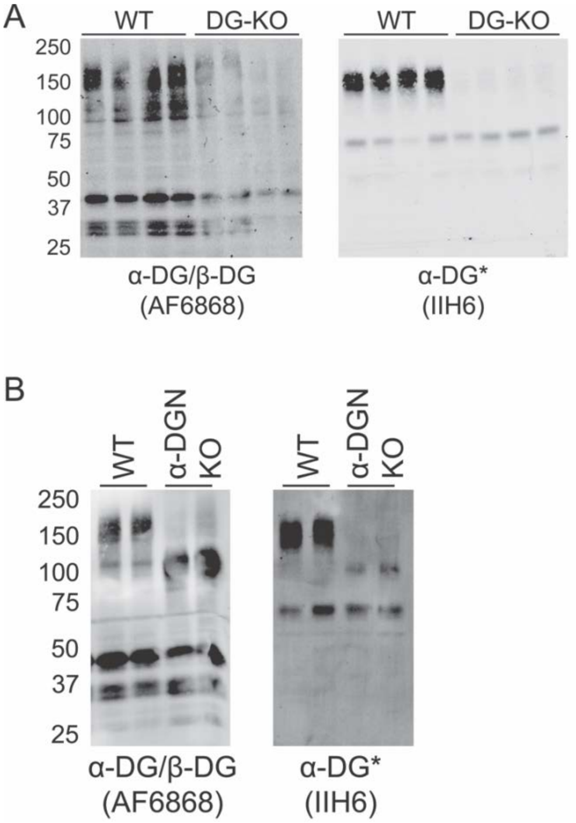
A
WT
DG-KO
250
150
100
75
50
37
25
α-DG/β-DG
(AF6868)
α-DG*
(IIH6)
B
WT
α-DGN
KO
α-DG/β-DG
(AF6868)
α-DG*
(IIH6)

Fig. S5. Mice that lack DG or α-DGN exhibit higher viral titers in the lungs after IAV infection.

A. Absolute data for titers of PR8 virus in whole lung tissue of control (WT) mice and those that lack DG (DG-KO) on day 4 post infection. Left graph shows experiment 1; right graph shows experiment 2. Each dot represents an individual mouse. TCID50 = 50% tissue culture infective dose.

B. Absolute data for titers of PR8 virus in whole lung tissue of control (WT) mice and those that lack α-DGN (α-DGN-KO mice) on day 4 post infection. Left graph shows experiment 1; right graph shows experiment 2. Each dot represents an individual mouse. TCID50 = 50% tissue culture infective dose.

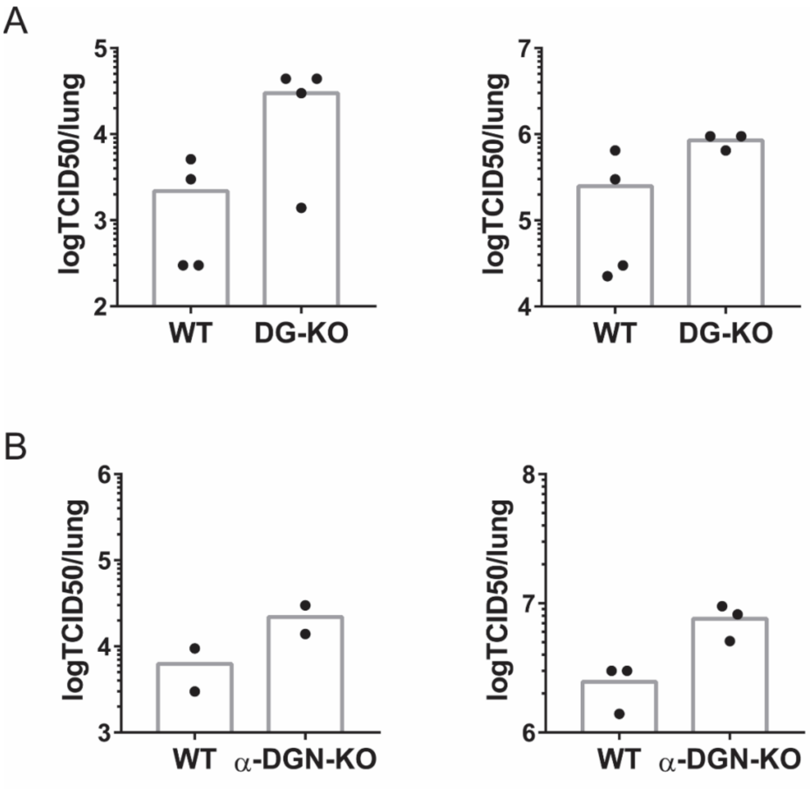

Fig. S6. Overexpression of α-DG reduces viral titers in the lungs after IAV infection.

A. α-DGN levels in the BAL fluid of C57Bl/6 mice that were infected two weeks prior with an adenovirus encoding wild-type DG (DG) or an adenovirus encoding a mutated form of DG in which the N-terminus cannot be cleaved (DGR312A). Each dot represents an individual mouse.

B. Absolute data for titers of PR8 virus in whole lung tissue of C57Bl/6 mice on day 4 post infection. Two weeks prior to infection, mice were infected with an adenovirus encoding wild-type DG (DG) or a mutated form of DG in which the N terminus cannot be cleaved (DGR312A). Left graph shows experiment 1; right graph shows experiment 2. Each dot represents an individual mouse. TCID50 = 50% tissue culture infective dose.

A

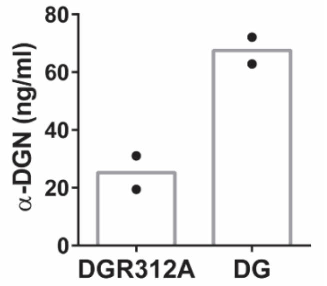

B

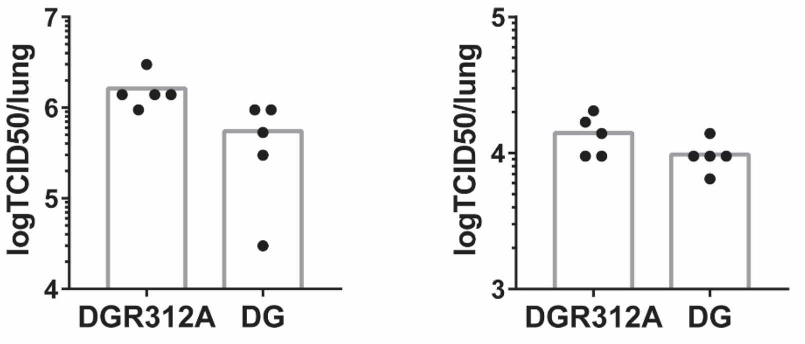

Fig. S7. Overexpression of α-DGN reduces viral titers in the lungs after IAV infection.

A. α-DGN levels in the BAL fluid of C57Bl/6 mice that were infected two weeks prior with a GFP-expressing adenovirus or HA-tagged α-DGN-GFP-expressing adenovirus. Each dot represents an individual mouse.

B. Absolute data for titers of PR8 virus in whole lung tissue of C57Bl/6 mice on day 4 post infection. Two weeks prior to infection, mice were infected with a GFP-expressing adenovirus or an HA-tagged α-DGN-GFP-expressing adenovirus. Left graph shows experiment 1; right graph shows experiment 2. Each dot represents an individual mouse. TCID50 = 50% tissue culture infective dose.

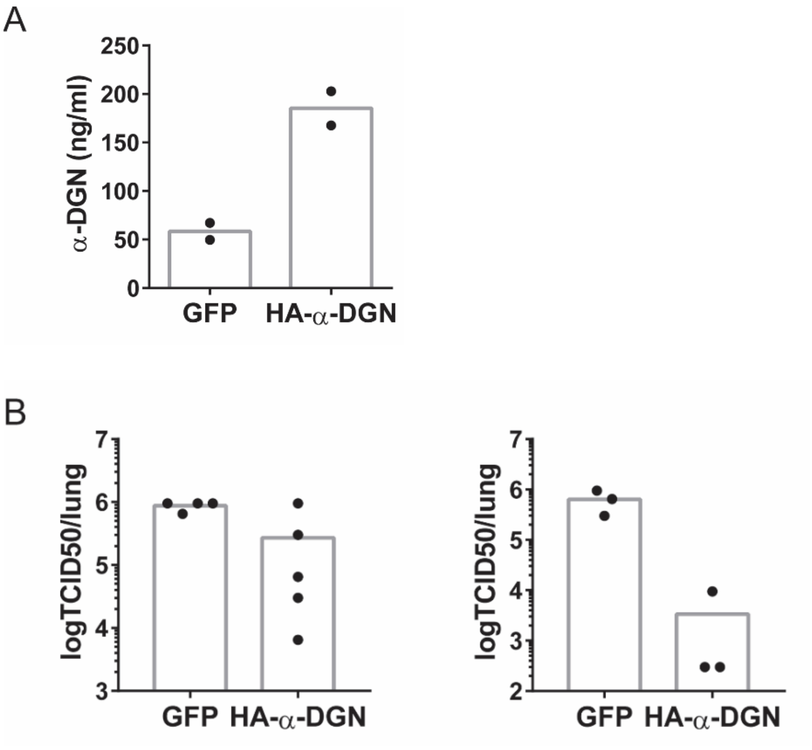

Fig. S8. Treatment with recombinant α-DGN reduces viral titers in the lungs after IAV infection.

A. Absolute data for titers of PR8 virus in whole lung tissue of C57Bl/6 mice on day 4 post infection. Mice were treated with the elution buffer imidazole or recombinant His-tagged α-DGN 1 day prior to infection and on days 1 and 3 post infection. Left graph shows experiment 1; right graph shows experiment 2. Each dot represents an individual mouse. TCID50 = 50% tissue culture infective dose.

B. Absolute data for titers of PR8 virus in whole lung tissue of C57Bl/6 mice on day 4 post infection. Mice were treated with the elution buffer imidazole or recombinant His-tagged α-DGN on day 1, 2, and 3 post infection. Left graph shows experiment 1; right graph shows experiment 2. Each dot represents an individual mouse. TCID50 = 50% tissue culture infective dose.

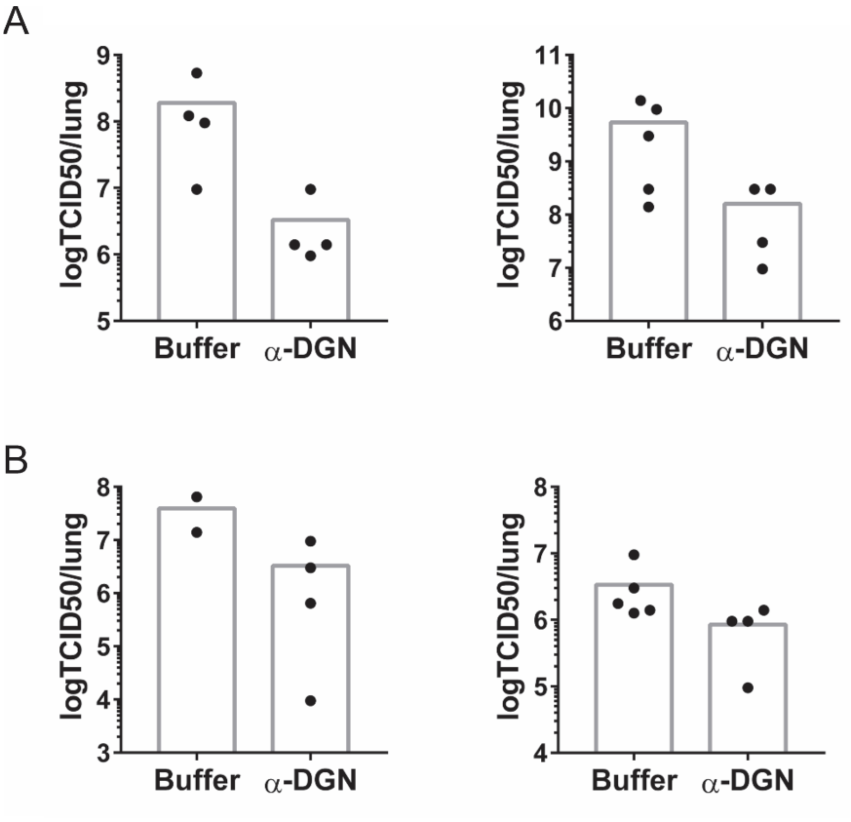

Supplement: Supplementary File [file pnas.1904493116.sapp.pdf]
